# External Validation of Dementia Risk Prediction Models: Towards evidence‐based implementation

**DOI:** 10.1002/alz70860_101969

**Published:** 2025-12-23

**Authors:** Blossom CM Stephan, Jacob Brain, Tanya Buchanan, Claire V Burley, Elissa Burton, Jennifer Dunne, Bronwyn Myers, Serena Sabatini, William Stephan, Eugene Yee Hing Tang, Kaarin J. Anstey, Mario Siervo

**Affiliations:** ^1^ Curtin University, Perth, Western Australia, Australia; ^2^ The University of Adelaide, Adelaide, SA, Australia; ^3^ University of Nottingham, Nottingham, Nottinghamshire, United Kingdom; ^4^ Dementia Australia, North Ryde, NSW, Australia; ^5^ Brightwater Research Centre, Perth, Western Australia, Australia; ^6^ South African Medical Research Council, Cape Town, Western Cape province, South Africa; ^7^ University of Cape Town, Cape Town, Western Cape province, South Africa; ^8^ University of Surrey, Guildford, Surrey, United Kingdom; ^9^ University of Barcelona, Barcelona, Catalonia, Spain; ^10^ Newcastle University, Newcastle, Newcastle upon Tyne, United Kingdom; ^11^ University of New South Wales, Sydney, NSW, Australia

## Abstract

**Background:**

Increasing dementia case numbers globally necessitates accurate and valid prediction tools for early intervention and prevention. Although over 100 different dementia prediction models exist none are endorsed for clinical use. With so many distinct models, it is difficult to make recommendations on which model should be selected for use. External validation – the assessment of model performance in populations distinct from the sample they were developed in – is critical for establishing utility and generalisability. Therefore, we undertook an umbrella review and meta‐analysis to evaluate the predictive performance of externally validated dementia prediction models.

**Method:**

We synthesised results from our three published systematic reviews on dementia risk prediction model development and testing, covering all literature from inception to mid‐2023. We also undertook an updated literature search (November 2024). Included studies were population‐based cohorts that evaluated predictive accuracy (e.g., c‐statistic) for an externally validated dementia prediction model. Meta‐analysis was conducted for models externally validated in ≥10 independent studies.

**Result:**

Out of 39 external validation studies, three models have been independently validated in ≥10 studies including the Brief Dementia Screening Indicator (BDSI), the Cardiovascular Risk Factors, Ageing and Dementia risk score (CAIDE) and the Genetic Risk Score‐19 (GRS‐19). Model validation has been exclusively undertaken in high and middle‐income countries. The meta‐analysis results show that the BDSI (pooled c‐statistic=0.72; 95%CI: 0.69‐0.75; I^2^=0.87; *n* = 13 external validations) and GRS‐19 (pooled c‐statistic=0.76; 95%CI: 0.74‐0.79; I^2^=0.81; *n* = 10 external validations), had reasonable predictive accuracy for dementia. In contrast, the CAIDE score showed poor accuracy (pooled c‐statistic=0.60; 95%CI: 0.55‐0.65; I^2^=0.95; *n* = 12 external validations). Limited transportability and heterogeneity in the results is likely due to methodological differences across studies, for example in sample age distribution and duration of follow‐up.

**Conclusion:**

With further real‐world testing, dementia risk prediction models that demonstrate reasonable external validity could be implemented in clinical settings to support early risk identification and preventative planning. Moving forward, research should evaluate the clinical impact and cost‐effectiveness of dementia risk screening, particularly in diverse populations and low/middle‐income countries, to optimize early detection and prevention efforts.

